# Transcriptome Profiling Reveals the Molecular Regulatory Network in Response to Salt Stress in Intervarietal Hybrids of *Chrysanthemum* × *morifolium* ‘Yannong Jinxia’ × ‘Yannong Qiujin’

**DOI:** 10.3390/biology15191703

**Published:** 2026-09-24

**Authors:** Xintong Li, Beining Zhang, Jiayi Zhou, Yansong Wang, Zimeng Li, Chunxin Dong, Hongbo Liu, Yufeng Cong, Li Zhao, Ri Gao

**Affiliations:** 1 College of Agricultural, Yanbian University, Yanji 133002, China; 2 Yanbian Province Forestry Research Institute, Yanji 133001, China

**Keywords:** ground cover chrysanthemum, hybridization, physiology, transcriptomics, salt stress

## Abstract

Salinity stress is a major abiotic factor that significantly constrains plant growth and development. Hybridization within the genus *Chrysanthemum* produces hybrid offspring exhibiting salt tolerance levels that are intermediate between those of the parental. However, the molecular mechanisms underlying this phenomenon remain unreported. In this study, the salt-sensitive chrysanthemum variety ‘Yannong Jinxia’ (JX) and the salt-tolerant chrysanthemum variety ‘Yannong Qiujin’ (QJ) were selected as parents for hybridization, aiming to elucidate the molecular basis of this intermediate salt tolerance. The hybrid offspring ‘Yannong Xiutang’ (XT) were verified using SRAP-PCR. After salt stress treatment, physiological indicators—including antioxidant enzyme activity and malondialdehyde (MDA) content—were measured in both the parents and the offspring. Their differential responses to salt stress were systematically compared by integrating transcriptome sequencing with KEGG enrichment analysis. Additionally, the expression patterns of key genes in the SOS signaling pathway were analyzed. Results showed that under salt stress, the hybrid XT exhibited intermediate levels of antioxidant enzyme activity and MDA accumulation relative to its parents. Transcriptomic analysis revealed distinct pathway enrichments: comparisons between QJ and XT highlighted differences in galactose metabolism, whereas JX vs. XT showed specific enrichment in pathways such as isoflavonoid biosynthesis and fatty acid elongation. Additionally, the expression of the SOS pathway gene in XT was found to be intermediate between the two parental lines. This study elucidates the molecular regulatory network associated with salt tolerance in hybrid offspring of ground cover chrysanthemum, laying a theoretical foundation for the development of salt-tolerant chrysanthemum germplasm.

## Simple Summary

Soil salinization is intensifying worldwide, threatening the sustainable cultivation of horticultural crops. Many chrysanthemum cultivars with high ornamental value exhibit poor salt tolerance. Therefore, developing novel chrysanthemum cultivars that combine high ornamental value with salt tolerance is of significant practical importance. In this study, the chrysanthemum cultivar ‘Yannong Qiujin’ (with superior ornamental traits) was crossed as the female parent with the highly salt-tolerant cultivar ‘Yannong Jinxia’ as the male parent. The hybrid progeny exhibited morphological traits similar to those of the female parent, but demonstrated significantly greater salt tolerance than the female parent. Transcriptome analysis showed that differentially expressed genes between the hybrid progeny and the female parent were mainly enriched in the galactose metabolism pathway. This study revealed the molecular regulatory network controlling salt tolerance in ground cover chrysanthemum hybrid progeny, offering a theoretical foundation for the development and breeding of salt-tolerant chrysanthemum germplasm.

## 1. Introduction

The global extent of saline–alkali land is estimated to cover approximately 1 billion hectares, accounting for roughly 7% of the total terrestrial area. This type of land is widely distributed across arid, semi-arid, and semi-humid regions on all continents [1]. As one of the countries with an extensive area of saline–alkali land in the world, China’s saline–alkali land is primarily distributed across the northeastern, northern, and northwestern inland regions, as well as the coastal areas north of the Yangtze River [2]. However, with the intensification of global warming, the evaporation of soil moisture has markedly increased, leading to the continuous expansion of saline–alkali land. At the same time, the issue of secondary salinization under facility cultivation has become progressively more pronounced, and salt stress has emerged as one of the critical abiotic stress factors constraining crop production. The damage induced by salt stress to plants primarily occurs through pathways such as osmotic stress, ionic toxicity, and oxidative stress [3]. The high concentration of salt in the soil induces elevated osmotic pressure, which impedes water absorption by plant roots. In severe cases, water may even flow out of the plant and back into the soil, leading to a physiological drought-like condition characterized by water deficit in plants. Plants exhibit symptoms such as leaf wilting and curling, stunted growth, and stomatal closure, all of which inhibit photosynthesis [4]. Consequently, due to the reduced synthesis of organic matter, plants exhibit slowed growth or a dwarfed phenotype. Furthermore, when plants absorb excessive amounts of harmful ions such as Na^+^ and Cl^−^, this disrupts the cellular ion balance, thereby inducing ion toxicity. For instance, the substitution of Na^+^ for K^+^ disrupts the activities of enzymes such as ATPase and photosynthetic enzymes, while the accumulation of Cl^−^ induces damage to chloroplast structure, causing chlorosis and scorching of leaves [5]. In addition, a high-salt environment inhibits the absorption of essential nutrients such as K^+^, Ca^2+^, and Mg^2+^ by the root system, triggering nutrient deficiency symptoms, such as stunted growth due to potassium deficiency and yellowing of leaves due to magnesium deficiency, which disrupts the nutritional balance [6].

Under salt stress, the production and scavenging of reactive oxygen species (ROS, such as H_2_O_2_ and O^2−^) within plant cells become imbalanced, leading to oxidative stress. This results in damage to the cell membrane structure, increased membrane permeability, and leakage of cellular contents, including malondialdehyde (MDA). Simultaneously, the activities of antioxidant enzymes are suppressed, further exacerbating oxidative damage [7]. During long-term evolution, plants have developed adaptive strategies in response to salt stress, which primarily include enhancing the activity of antioxidant enzymes, pro-moting the synthesis of osmotic regulatory substances, and maintaining ion homeostasis [8]. Under salt stress, plants enhance the activities of antioxidant enzymes, including su-peroxide dismutase (SOD), peroxidase (POD), and catalase (CAT), thereby establishing an efficient mechanism for scavenging ROS. Under salt stress, the halophyte Limonium sinense activates its antioxidant system by upregulating CAT, SOD, and POD, thereby effectively scavenging ROS to maintain normal growth under high-salinity conditions [7]. Mittova et al. [8] reported that the activities of SOD, POD in the tomato antioxidant system increased significantly with increasing salt concentration. Moreover, soil water potential decreases significantly, markedly impairing plant water uptake capacity under high-salinity stress. As an adaptive response to salt stress, plants synthesize soluble osmoregulatory substances such as proline (Pro), betaine, and soluble sugars, which effectively lower cellular osmotic potential, thereby maintaining water uptake capacity and alleviating ionic toxicity. Betaine is synthesized by choline monooxygenase and betaine aldehyde dehydrogenase. It effectively reduces cellular osmotic pressure in the cytoplasm without interfering with enzyme activity and can counteract the inhibitory effects of Na^+^ on enzymes [9]. Soluble sugars not only serve as an energy source for plants but also function as osmoprotectants, playing significant roles in various physiological and biological processes [10].

Among the molecular mechanisms of plant salt tolerance, the SOS (Salt Overly Sensitive) signaling pathway is one of the most extensively studied regulatory pathways. In the SOS signaling pathway, the calcium-binding protein SOS3 specifically interacts with the protein kinase SOS2 to activate its kinase activity [6]. The activated SOS2 phosphorylates the plasma membrane-localized Na^+^/H^+^ antiporter SOS1, thereby promoting the efflux of Na^+^ from the cytoplasm to the extracellular space and reducing the intracellular Na^+^ concentration [11]. Studies have demonstrated that the overexpression of the SOS1 gene in rice and maize significantly enhances the salt tolerance of these plants [12,13]. Recent advances have expanded chrysanthemum salt-tolerance research from single-gene validation to multi-omics and epigenetic analyses. Transcriptomic and metabolomic comparisons of autopolyploid chrysanthemums revealed that ABA- and JA-mediated networks distinguish tolerant genotypes [14]. Epigenetically, ABA–SnRK2.2–ABF1/BRM signaling dynamically remodels *H3K4me3* deposition at the *CmHSFA4* promoter, directly linking chromatin state to salt-responsive transcription [15]. Meanwhile, *CmDOF2* [16] and *CiHY5* [17] alleviate ROS accumulation by enhancing antioxidant enzyme activity. Proteomics further confirms redox homeostasis and ion transport as central to salt response [18]. Despite these advances, salt tolerance in chrysanthemum interspecific hybrids remains poorly studied.

Plant hybrid progeny may exhibit enhanced salt tolerance. In the rice hybridization process, ‘indica’ was used as the paternal parent and ‘japonica’ as the maternal parent. The yield of the hybrid offspring increased significantly compared to that of the parents, and salt tolerance also improved. Through transcriptome analysis, it was found that differentially expressed genes in hybrid rice were primarily enriched in the ethylene, auxin, gibberellin, and antioxidant metabolism pathways [19]. The hybrid ‘CAFDS2’ was obtained by crossing poplar ‘CAFDF’ with ‘CAFDM’. After salt treatment, the expression levels of genes related to the PSII and PSI reaction centers and ATP synthase in the hybrid offspring were significantly higher than those in the parents. This effectively alleviated the damage to the electron transport chain and maintained the energy supply for plant growth and development, thereby enhancing the salt tolerance of the hybrid offspring [20].

From the above, it is apparent that there may be differences in the salt tolerance mechanisms of hybrid offspring resulting from different plant crosses. Crosses between *C. dichrum* and *C. nankingense* exhibited phenotypic variation ranging from 8.81% to 55.78%. Meanwhile, a total of 339 significant quantitative trait nucleotides (QTNs) and 40 suggestive QTNs were identified [21]. Researchers performed interspecific hybridization between *Chrysanthemum* ‘Maoyan’ and *Artemisia japonica* Thunb. The salt tolerance of the hybrid offspring was intermediate between that of the two parents, yet significantly higher than that of the female parent [22]. The salt tolerance of the offspring resulting from the hybridization of *Crossostephium chinense* and *C. lavandulifolium*, as well as the offspring from the hybridization of *C. grandiflorum*, *Artemisia vulgaris*, and *C. chinense*, was significantly enhanced following salttreatment [23]. The hybrid progeny from the cross between ground cover chrysanthemum cultivars ‘Yannong Hongxue’ (female parent) and ‘Yannong Chenghuang’ exhibited significantly higher expression levels of *CmSOS1* and *CmHKT* compared with the female parent, concomitant with enhanced salt tolerance [24]. Although several salt-tolerant varieties have been developed through hybridization in the *Chrysanthemum*, the molecular mechanisms underlying their salt tolerance remain largely unelucidated.

Ground Cover Chrysanthemum (*Chrysanthemum* × *morifolium* ‘Ground Cover Chrysanthemum’) is a perennial herb of the genus *Chrysanthemum* in the Asteraceae family. It is characterized by a diverse range of flower colors, a long flowering period, and remarkable environmental adaptability. Due to its high ornamental value, it has been widely applied in landscaping projects and as cut flowers. During the cultivation process, secondary soil salinization seriously affects the growth and quality of chrysanthemums, resulting in considerable economic losses [25]. Therefore, in this study, ‘Yannong Jinxia’ and ‘Yannong Qiujin’ were used as parental materials for hybridization, and molecular markers were employed to identify the hybrid progeny. The hybrid progeny were subjected to salt treatment, and their phenotypic changes were observed. Additionally, the activities of antioxidant enzymes and other relevant indicators were detected to comprehensively evaluate their salt tolerance. Furthermore, transcriptome-based analysis was performed to elucidate the molecular mechanisms underlying salt tolerance in the hybrid progeny. These findings, derived from transcriptomic profiling, provide a theoretical basis for breeding novel salt-tolerant ground cover chrysanthemum.

## 2. Materials and Methods

### 2.1. Ground Cover Chrysanthemum ‘Yannong Jinxia’ × ‘Yannong Qiujin’ Hybridization

The ‘Yannong Jinxia’, characterized by a neat growth form and light pink flowers, was selected as the male parent, while ‘Yannong Qiujin’, with strong salt tolerance, was chosen as the female parent. Both chrysanthemum parental cultivars used in this study were purchased from Lüxin Flower and Horticulture Co., Ltd., Shenbei New District (Shenyang, China), and all parental seedlings were propagated via rooted cuttings. Both varieties were cultivated in the teaching base of Yanbian University (Yanji, China) in the spring season. In late September, when the female parent ‘Yannong Qiujin’ reached the early flowering stage and had not yet released pollen, all tubular florets were carefully excised using fine-tipped tweezers, and the apical 0.5–0.8 cm of each ray floret was removed with sterile scissors. Once the operation was complete, promptly place a sulfur-impregnated paper bag (8 cm × 12 cm) over the flowers. After 2 to 3 days, when the stigmas of the maternal plant had developed a “Y”-shaped morphology, pollen was collected from the paternal cultivar ‘Yannong Jinxia’ for artificial pollination. The artificial pollination procedure was carried out following the method described by Shan et al. [24]. A total of 10 plants of the ‘Yannong Jinxia’ were used, with 15 inflorescences per plant, yielding approximately 150 inflorescences for crossing. For the ‘Yannong Qiujin’, 10 plants served as the pollen source. After 40 to 50 days, when the flowers had withered and the seeds matured, the seeds were promptly harvested and stored at low temperature.

### 2.2. SRAP Analysis of ‘Yannong Jinxia’ × ‘Yannong Qiujin’ Progeny

Leaves were collected from parental plants and their hybrid offspring. Samples were thoroughly rinsed with sterile distilled water to remove surface contaminants and then gently blotted dry using sterile paper towels. Genomic DNA was subsequently extracted following the manufacturer’s protocol for the TIANGEN DNA extraction kit (TIANGEN Biotech, Beijing, China). The concentration and purity of the extracted DNA were evaluated by 1.0% (*w*/*v*) agarose gel electrophoresis and spectrophotometric analysis using a NanoDrop™ One microvolume UV–Vis spectrophotometer (Merinton Instrument Inc., Ann Arbor, MI, USA). DNA concentrations of all parental and hybrid samples were normalized to 200 ng/μL prior to Sequence-Related Amplified Polymorphism (SRAP) PCR amplification. The total volume of the SRAP reaction system was 25 μL, consisting of 2 μL of DNA, 1 μL of primers, 1.5 μL of dNTP mixture, 2.0 μL of Mg^2+^, 0.2 μL of rTaq, and 2.5 μL of 10 × PCR buffer and ddH_2_O to fill the remaining volume. The primer sequences are shown in Table S2. The PCR amplification program was as follows: initial denaturation at 94 °C for 4 min; 4 cycles of denaturation at 94 °C for 1 min, annealing at 35 °C for 1 min, and extension at 72 °C for 2 min; 35 cycles of denaturation at 94 °C for 1 min, annealing at 50 °C for 1 min, and extension at 72 °C for 2 min; and a final extension at 72 °C for 10 min. After electrophoresis of genomic DNA (2% agarose gel, 120 V, 30 min), digital images were captured using a ChemiDoc™ XRS+ system with UV transillumination (Model GelView 5000Plus, BLTPhoton Technology Co., Ltd., Guangzhou, China). Gray-scale analysis was performed using Image Lab™ software (version 6.1), and the number of bands was quantified. Subsequently, Shannon’s information index, Nei’s genetic diversity index, and the proportion of polymorphic bands were analyzed using PopGene 32 software. Data reproducibility was verified through three independent technical replicates.

### 2.3. Morphological and Hybrid Dominance Analysis of ‘Yannong Jinxia’ × ‘Yannong Qiujin’ Progeny

During the flowering period, the morphology of the hybrid offspring and parents of ‘Yannong Jinxia’ × ‘Yannong Qiujin’, including plant height, canopy width, leaf length and width, flower color, flower pedicel diameter, number of tubular flowers, and number of lamellar flowers, were investigated and statistically analyzed. Morphological investigation was conducted according to the reference provided by Kumar [26]. Plant height was measured as the vertical distance from the base of the stem to the highest point of the plant; The leaf length and width were determined by measuring the maximum length and width of the third leaf from the top. After data collection was completed, mid-parental dominance (Hm) and mid-parental dominance rate were further calculated.

Medium Parental Value (MPV) = (Paternal Trait Value + Maternal Trait Value)/2; Neutrophilic dominance (H_m_) = (Mean of traits of cross progeny (Fm) − MPV); Neutrophilic dominance rate (RH_m_) = Hm/MPV.

### 2.4. ‘Yannong Jinxia’ × ‘Yannong Qiujin’ Progeny Salt Treatment and Oxidation Reaction

The root suckers of the offspring and parents of ‘Yannong Jinxia’, ‘Yannong Qiujin’ were collected for cutting propagation. The substrate was prepared by mixing humus soil and perlite at a 1:1 volume ratio. The growth conditions were maintained at 25 ± 2 °C with a 14 h daily photoperiod. When the seedlings have grown to 5–6 leaves, they are transplanted into a 0.5-fold diluted Hoglund’s nutrient solution for a 1-week pre-culture to acclimate them to the hydroponic environment. After the pre-culture, eighteen plants of each genotype (parental lines and hybrid progeny) were subjected to 200 mM NaCl treatment for 0 h, 1 h, 2 h, 4 h, 6 h and 8 h, respectively, to investigate the early physiological features of the salt stress response. Three to four leaves from the top of each plant were collected for the oxidation reaction analysis. Three biological replicates were set up for each treatment. The activity of POD was determined using the guaiacol method, the activity of SOD was measured via the nitroblue tetrazolium photoreduction method, and the activity of CAT was assessed by potassium permanganate titration [27,28,29]. Additionally, the Pro content was quantified using the acidic ninhydrin colorimetric method, while the MDA content was determined through the thiobarbituric acid method [30,31]. Relative electrolyte leakage (REL) in leaves was quantified using the conventional boiling water conductivity method. Approximately 0.1–0.2 g of salt-treated leaf samples were immersed in 10 mL of deionized water and incubated at 25 °C with shaking for 12 h. The electrical conductivity of the extract (EC_1_) was measured using a conductivity meter. The samples were then subjected to a boiling water bath for 15 min to rupture cell membranes completely. After cooling to room temperature, the electrical conductivity (EC_2_) was determined. Relative electrical conductivity (REC, %) was calculated as REC (%) = [(EC_1_ − EC_0_)/(EC_2_ − EC_0_)] × 100%, where EC_0_ represents the electrical conductivity of blank deionized water. Soluble sugar content was measured by the anthrone–sulfuric acid method following Zhao et al. [32]. Chlorophyll content was determined using the 95% ethanol extraction method, and the absorbance of the extract was read at 645 and 663 nm; chlorophyll a, chlorophyll b, and total chlorophyll concentrations were calculated using the equations of Lichtenthaler and Wellburn [33]. All physiological data are presented as mean ± SD (n = 3 biological replicates). One-way analysis of variance (ANOVA) followed by Tukey’s honestly significant difference (HSD) test (*p* < 0.05) was conducted. Detection of ROS distribution using DAB (3,3′-Diaminobiphenylamine) and NBT (Nitroblue Tetrazolium) histochemical staining methods: After treating the hybrid offspring with salt, the leaves were, respectively, immersed in a 0.1% NBT solution (pH 7.8, containing 10 mM NaN_3_) and a 1 mg/mL DAB solution (pH 3.8, containing 0.05% Tween-20) in the dark at room temperature for 12–16 h. Following incubation, the leaves were decolorized in 95% ethanol in an 80 °C water bath 2–3 times (each for 15–20 min) until the leaf background became transparent and the color spots were clearly visible. Finally, the staining results were photographed and recorded under a stereomicroscope.

### 2.5. Determination of Na^+^ and K^+^ in ‘Yannong Jinxia’ × ‘Yannong Qiujin’ Progeny After Salt Treatment

As previously described [34], ground cover chrysanthemum leaves subjected to salt treatment were placed in a constant-temperature oven and dried at 120 °C for 2 h. After drying, the samples were ground into a fine powder, and 0.10–0.20 g of the powdered plant material was used for subsequent analyses. Transfer the prepared samples into a 100 mL digestion tube. Add a small volume of deionized water to moisten the sample matrix, followed by the addition of 5 mL of concentrated H_2_SO_4_. Gently shake the tube to ensure complete and uniform contact between the samples and H_2_SO_4_. Following overnight standing, the digestion tube is placed on the digestion furnace for heating and digestion until the solution becomes colorless or transparent. Upon completion of digestion, the solution is filtered, and the clear filtrate is collected for analysis. The concentrations of Na^+^ (sodium ions) and K^+^ (potassium ions) are determined using a WGH-6450 flame photometric spectrophotometer (LICHEN, Shanghai, China).

### 2.6. Transcriptome Analysis of ‘Yannong Jinxia’ × ‘Yannong Qiujin’ Progeny After Salt Treatment

The 5–6-leaf hybrid offspring of ‘Yannong Jinxia’ × ‘Yannong Qiujin’ and their parental seedlings were subjected to 200 mM NaCl treatment for 4 h, after which leaves were collected for transcriptome sequencing. Three biological replicates were performed for each treatment. Total RNA was extracted from the frozen samples using the TransZol Plant Kit (TIANGEN, Beijing, China) strictly following the manufacturer’s instructions. The integrity and quality of the RNA evaluated through agarose gel electrophoresis, after which the qualified RNA samples were sent to BioMarker Biotechnology Co., Ltd., Beijing, China for sequencing. The paired-end transcriptome sequencing was performed on the Illumina HiSeq 4000 platform. The raw data (Raw Reads) were processed using Cutadapt (v4.4) to remove low-quality sequences and adapter contaminants, yielding high-quality Clean Reads. Based on *HISAT2*, a reference genome index was constructed, and the Clean Reads were aligned to the *C. lavandulifolium* genome (https://cgd.njau.edu.cn/asteraceae/browse/genomePage/Cl (accessed on 23 March 2025)) for analysis. The gene expression levels were quantified using the FPKM (Fragments Per Kilobase Million) normalization method, and differential expression analysis was performed using DESeq2. Differentially expressed genes (DEGs) were identified based on the criteria of |log2(Fold Change)| ≥ 1.5 and an adjusted *p*-value (padj) < 0.05. Finally, the DEGs were analyzed for Gene Ontology (GO) functional enrichment and KEGG pathway annotation using the R packages topGO (version 2.44.0) and clusterProfiler (version 4.4.4). The significance thresholds were set as FDR < 0.01 for GO terms and *p* < 0.01 for KEGG pathways.

### 2.7. Expression Levels of Salt Tolerance-Related Genes

The relative expression levels of *CmGIP1*, *CmFRL3*, *CmXRN4*, *CmRKF1*, *CmGAF1*, *CmWRKY70*, *CmBAK1*, *CmSAPK3*, *CmSIS8*, *CmM3KE1*, *CmbHLH93* and *CmNLP7* in the leaves of the hybrid offspring and their parents were detected by real-time fluorescence quantitative PCR (qRT-PCR). The reaction system consisted of 2 × SuperReal PreMix Plus 10 μL, cDNA 5 μL, 50×ROX Reference Dye^(Δ)^ 0.4 μL, Primer F 1 μL, Primer R 1 μL, RNase-free ddH_2_O 2.6 μL. Reaction program: 95 °C for 2 min; 95 °C for 5 s, 55 °C for 10 s, and 72 °C for 15 s for 40 cycles, and the melting curve was selected from 60 °C to 95 °C. The internal reference gene was *CmEF1α*. The primer sequences for the salt tolerance-related genes are listed in Table S2. Each treatment was performed in triplicate. Data analysis was conducted using the 2^−ΔΔCT^ method.

### 2.8. Data Analysis

Data were organized and visualized using GraphPad Prism 8 (GraphPad Software, San Diego, CA, USA). All data are presented as mean ± standard deviation (SD) (n = 3 biological replicates). Prior to statistical analysis, data normality was assessed using the Shapiro–Wilk test, and homogeneity of variances was evaluated using Levene’s test. One-way analysis of variance (ANOVA) followed by Tukey’s honestly significant difference (HSD) post hoc test was performed using SPSS Statistics 26.0 (IBM Corp., Armonk, NY, USA). Statistical significance was set at *p* < 0.05.

## 3. Results and Analysis

### 3.1. Authentication Identification and Polymorphism Analysis of Hybrid Progeny

To verify the authenticity of the hybrid progeny ‘Yannong Jinxia’ × ‘Yannong Qiujin’ and to investigate their genetic polymorphism, this study employed the SRAP-PCR technique. Specific primers were designed for PCR amplification of genomic DNA from the hybrid offspring and their parental lines. The results demonstrated that the amplified bands were clear, highly reproducible, and exhibited abundant polymorphic loci, making them suitable for subsequent data analysis. A total of 231 bands were generated from the hybrid offspring and their parental lines using 13 primer pairs. The number of bands amplified per SRAP primer combination ranged from 3 to 10, with an average of 5.93 bands per combination. In the SRAP-PCR amplification with the ME25 + EM2 and ME25 + EM3 primer combinations, the male parent and the hybrid offspring exhibited characteristic common bands. In contrast, the ME25 + EM4, ME25 + EM5, and ME25 + EM6 primer combinations specifically amplified common bands in both the female parent and the hybrid offspring (Figure 1). The Shannon information index ranged from 0 to 0.69, with a mean value of 0.52, whereas the Nei gene diversity index ranged from 0 to 0.50, with a mean value of 0.37. The percentage of polymorphic loci observed varied between 66.67% and 100% (Table S4).

### 3.2. Morphological of the Hybrid Progeny and Hybrid Advantage

An investigation into the morphology of the ‘Yannong Jinxia’ × ‘Yannong Qiujin’ hybrid progeny at full bloom revealed that their plant height, crown width, inflorescence diameter, ligule number, and ligule length were 36.83 cm, 55.50 cm, 5.47 cm, 33, and 2.38 cm, respectively, and exceeded those of the parents. The mid-parental dominance rate ranged from 18.72% to 36.30%, indicating the superior performance of the hybrid progeny compared to both parents in these traits. The leaf length, leaf width, and number of ligule flowers were 4.13 cm, 2.55 cm, and 50, respectively, all of which were lower than those of the parents. The mid-parental dominance values were negative, indicating a decrease in hybrid dominance for these traits in the progeny. The hybrid offspring display an orange-red flower color (R: 206, G: 64, B: 41) with a double-layered petal structure. Their coloration is more similar to that of the male parent plant, while the morphology of the tubular flowers closely resembles that of the female parent plant (Figure 2, Table S1).

### 3.3. Phenotypic and Oxidative Responses of Hybrid Progeny Under Salt Stress

Upon subjecting ‘Yannong Jinxia’, ‘Yannong Qiujin’, and their hybrid offspring to salt stress treatment, it was observed that with the prolonged duration of salt stress, the overall wilting severity of the plants progressively increased. By 8 h, the degree of wilting was milder in the parental ‘Yannong Jinxia’ and its hybrid progeny than in the parental ‘Yannong Qiujin’ (Figure 3A). The hybrid progeny were subjected to salt stress treatment and subsequently stained with DAB and NBT for observing the color change. The results showed that with the increasing duration of salt treatment, the brownish-gray and blue spots on the leaves became progressively more severe. After being treated with salt for 8 h and stained with DAB, it was observed that the hybrid offspring and the female parent plant exhibited a paler brown color compared to the paternal plant. NBT staining revealed that the hybrid offspring and the maternal plant exhibited fewer blue spots than the paternal plant (Figure 3B). After 8 h of salt stress treatment, the activities of SOD, CAT, and POD, as well as the contents of Pro and soluble sugar in the maternal plants, reached the highest values, measuring 119 U·g^−1^·min^−1^, 25.08 U·g^−1^·min^−1^, 44.77 U·g^−1^·min^−1^, 0.047 μg·g^−1^, and 3.58 mg·g^−1^, respectively. The hybrid progeny exhibited the next highest values for these indicators (Figure 3C–G). Chlorophyll content gradually decreased with prolonged salt stress duration. After 8 h of treatment, the chlorophyll content in the maternal plants was 0.94 mg·g^−1^, significantly higher than that in the hybrid progeny and the paternal plants (Figure 3H). The relative electrolyte leakage and MDA content in the leaves of the paternal plants were 0.83% and 0.0048 μg·g^−1^, respectively, both significantly higher than those in the hybrid progeny and the maternal plants ‘Yannong Qiujin’ (Figure 3I,J). Furthermore, the Na^+^ and K^+^ contents in the hybrid offspring and parental plants were measured after salt stress treatment. The results showed that salt stress led to a decrease in K^+^ content and an increase in Na^+^ content across the plant samples. After 8 h of salt stress treatment, the paternal parent ‘Yannong Jinxia’ exhibited a Na^+^ content of 33.14 mg·g^−1^ and a Na^+^/K^+^ ratio of 1.13, both significantly higher than those in the hybrid progeny and the maternal parent ‘Yannong Qiujin’ (Figure 3K,M). In contrast, the K^+^ content in the paternal parent was 28.89 mg·g^−1^, which was significantly lower than that in the hybrid progeny and the maternal parent (Figure 3L).

### 3.4. Transcriptome Sequencing of Hybrid Progeny Under Salt Treatment

To evaluate the molecular characteristics of salt tolerance in the hybrid offspring and their parents, this study conducted RNA-Seq analysis on the samples. Nine cDNA libraries were constructed for the experiment, and paired-end sequencing was performed on these libraries. A total of 58.56 GB of Clean Data was generated. Each sample produced more than 6.15 GB of Clean Data, with a Q30 base exceeding 95.23%. The Clean Reads of each sample were aligned to the reference genome. The alignment efficiency ranged from 58.46% to 62.79%. Based on these alignment results, further analysis was performed for gene structure optimization. A total of 11,434 novel genes were identified, of which 4887 were functionally annotated (Table S5). Principal Component Analysis (PCA) was conducted to compare the gene expression patterns among the hybrid offspring, ‘Yannong Jinxia’, and ‘Yannong Qiujin’. The results revealed that PC1 (Principal Component 1) explained 40.35% of the variance between the hybrid and its parents, while PC2 accounted for 21.6% of the variation. (Figure 4A). These findings suggest that the gene expression patterns of the hybrid offspring and the parental plants exhibited differentiation in the principal component space. The correlation heatmap analysis revealed that the mean Pearson correlation coefficient for pairwise comparisons among the libraries was 0.829, with all pairwise correlation coefficients exceeding 0.785 (Figure 4B).

### 3.5. GO Functional Enrichment Analysis of DEGs in Hybrid Offspring

The volcano plot analysis identified 7085 differentially expressed genes (DEGs) in QJ vs. XT (3962 up-regulated and 3123 down-regulated), 6546 DEGs in JX vs. XT (2715 up-regulated and 3831 down-regulated), and 12,567 DEGs in JX vs. QJ (5744 up-regulated and 6823 down-regulated) (Figure 5A). The GO enrichment analysis graph indicates that the DEGs in the three groups exhibit significant enrichment in the three main categories: biological process (BP), cellular component (CC), and molecular function (MF). Specifically, BP is primarily enriched in “cellular process”, “metabolic process”, and “biological regulation”; CC shows significant enrichment in “cellular anatomical entity”, “intracellular components”, and “protein-containing complexes”; and MF is predominantly associated with “binding”, “catalytic activity”, and “transporter activity“ (Figure 5B–D). Furthermore, Venn diagram analysis identified 1069 DEGs common to all three groups (Figure 5E), suggesting that these genes may play a fundamental role in the common mechanism underlying the response of both hybrid offspring and parents to salt stress.

### 3.6. KEGG Pathway Enrichment Analysis of DEGs in Hybrid Offspring and Parental Plants

To further investigate the metabolic pathway regulatory mechanisms underlying the hybrid offspring’s response to salt stress, this study performed a KEGG pathway enrichment analysis on the identified DEGs. In the three comparisons of QJ vs. XT, JX vs. XT, and JX vs. QJ, it was found that genes associated with ribosome, carbon metabolism, plant hormone signal transduction, starch and sucrose metabolism, phenylpropanoid biosynthesis, carotenoid biosynthesis, and linoleic acid metabolism were significantly enriched. This suggests that these pathways may play a common regulatory role in the salt stress responses of both the hybrid offspring and their parents. Furthermore, compared to other groups, the QJ vs. XT comparison exhibited significant differences in gene enrichment within metabolic pathways such as galactose metabolism (Figure 6A). The enrichment of differential genes in metabolic pathways such as isoflavonoid biosynthesis and fatty acid elongation was only present in the JX vs. XT group (Figure 6B). Meanwhile, the enrichment of differential genes in pathways such as porphyrin and chlorophyll metabolism, and glycerophospholipid metabolism was also specifically observed in the JX vs. QJ group (Figure 6C).

### 3.7. Validation of Transcriptome Data

Twelve genes randomly selected for their association with the MAPK signaling pathway and ribosome pathway were validated by qRT-PCR analysis. After 4 h of salt treatment, the qRT-PCR results indicated that the expression levels of the EVM0040501, EVM0043879, EVM0055082 and EVM0035906 genes in hybrid progeny reached 9.82, 5.81, 11.97 and 6.28, respectively, among these groups, with the paternal cultivar ‘Yannong Jinxia’ exhibiting the lowest expression levels. These genes, including EVM0051158, EVM0063533, EVM0062893, EVM0067762, EVM0055469, EVM0056454, EVM0000280 and EVM0018769, exhibit the highest expression levels in ‘Yannong Xiutang’, whereas their expression levels were lower in ‘Yannong Jinxia’ (Figure 7). The relative expression levels of these genes are consistent with the trend of FPKM data in the transcriptome, indicating that the data reported of RNA-seq provides a reliable basis for subsequent analysis.

## 4. Discussion

As the global extent of soil salinization continues to increase, the development of new salt-tolerant crop varieties has become a core strategy for promoting sustainable agricultural development. Crossbreeding is a pivotal technique for developing novel salt-tolerant crop cultivars, and accurate identification of hybrid progeny constitutes a critical step in the crossbreeding process.

Currently, the commonly employed methods for this purpose include chromosome number detection and molecular marker technology. When the chromosome number of the hybrid offspring is consistent with that of the parents, molecular marker technology serves as the primary identification method. With the advancement of molecular biology technology. SRAP represents a novel PCR-based molecular marker technology developed by Li and Quiros [35]. This technology is characterized by simple operation, rapid detection, high polymorphism, excellent repeatability, and relatively low cost [36]. Researchers employed SRAP molecular markers to authenticate 84 progenies derived from eight *Stylosanthes guianensis* cross combinations, successfully identifying 68 true hybrids [37], and conducted the detection of presence/absence polymorphisms of specific markers and DNA bands at particular genomic loci, successfully performing genetic identification of 29 tree peony cultivars. This study revealed that characteristic common bands existed between the male plants and the hybrid plants, while specific common bands were also observed between the female plants and the hybrid progeny plants. This result further validated the genetic authenticity of the hybrid progeny plants. Furthermore, a phenotypic investigation of the hybrid offspring revealed that their plant height, canopy width, and inflorescence diameter were significantly greater than those of the parents, whereas their leaf length and width were smaller. Additionally, the flower color was more bright red. Overall, the hybrid offspring exhibited larger flower sizes with denser arrangements, more compact plants, and higher ornamental value compared to the parents. The hybrid progeny of *Dendranthema morifolium* variety ‘Zhongshanjingui’ and *Artemisia vulgaris* ‘Variegata’ not only enhanced ornamental value but also effectively inherited aphid resistance traits [38].

Under salt stress conditions, different chrysanthemum varieties exhibit differences in salt tolerance. Studies have demonstrated that varieties with stronger salt tolerance generally display higher antioxidant enzyme activities. These enzymes effectively scavenge ROS induced by salt stress, thereby alleviating oxidative stress and protecting cells from damage [39,40]. In contrast, varieties with lower salt tolerance exhibit insufficient antioxidant enzyme activities, leading to inefficient ROS removal and subsequent excessive accumulation of ROS within the cells, which causes severe cellular damage. Studies have shown that under salt treatment conditions, the activities of SOD, CAT, and POD in the seeding of chrysanthemum, tomato, and pear exhibited an increasing trend with increasing salt concentration, and the activity levels of each enzyme were significantly higher than those in the control group [41,42]. These findings indicate a relevant positive correlation between enhanced antioxidant enzyme activities and salt-tolerant phenotypes. This study demonstrates that after exposure to a salt concentration of 200 mmol/L for 8 h, the antioxidant enzyme activities in all three groups significantly increased. Notably, the enzyme activities of both the hybrid offspring and the maternal parent ‘Yannong Qiujin’ were higher than those of the paternal parent ‘Yannong Jinxia’. The results of DAB and NBT staining were consistent with the changes in antioxidant enzyme activity, suggesting that the extent of membrane lipid damage in the hybrid offspring was intermediate between the two parents but less severe than in the paternal parent. Salt stress can cause damage to the structural integrity of the cell membrane, leading to the leakage of electrolytes, and at the same time, it is accompanied by the accumulation of membrane lipid peroxidation product MDA. Therefore, both electrolyte leakage and MDA accumulation serve as reliable indicators for assessing plant salt tolerance [43,44]. In this experiment, the MDA content and relative electrolyte conductivity of the hybrid progeny were intermediate between those of the parental lines and lower than those of either parent, indicating that the extent of membrane damage in the hybrid progeny was less severe than in the paternal parent. Concurrently, qPCR analysis revealed that the relative expression levels of *CmSOD*, *CmCAT2*, *CmPRORP1*, *CmHKT1* and *CmCAT9* in the maternal and hybrid progeny were higher than those in the paternal parent. Taken together, these results showed that the salt tolerance of the hybrid progeny of ‘Yannong Jinxia’ × ‘Yannong Qiujin’was better than that of the parent ‘Yannong Jinxia’.

When plants are exposed to salt stress, Na^+^ and Ca^2+^ accumulate significantly within the cells. These ions not only induce ionic toxicity but also disrupt ionic homeostasis [45]. A critical mechanism for maintaining ion homeostasis in plants involves the efflux of Na^+^ from the cytoplasm, which is primarily mediated by Na^+^/H^+^ antiporters. The SOS regulatory pathway is an important way for plants to regulate the activity of Na^+^/H^+^ antiporters and maintain ionic homeostasis under salt stress [46]. This process facilitates the extrusion of excess Na^+^ from the cytoplasm into the apoplast, thereby reducing Na^+^ accumulation in the cytoplasm and enabling plants to maintain normal physiological functions under salt-stress conditions. Studies demonstrated that the maize *ZmSOS3*/*ZmSOS2* complex phosphorylates *ZmSOS1*, thereby activating its Na+-transporting activity [47]. Moreover, loss-of-function mutants of *ZmSOS1* exhibit salt-hypersensitive phenotypes. During salt stress responses, NHX proteins help maintain a lower intracellular Na^+^ concentration by either expelling Na^+^ absorbed from the roots or by isolating Na^+^ in the vacuoles [48]. In addition, 14-3-3 proteins, GIGANTEA protein (GI), ABA Insensitive 2 (ABI2) protein, phosphatidylinositol 4-kinase (PI4K) and BIN2 protein regulate the SOS pathway by modulating the activity of the SOS2 enzyme (Figure 8). After salt treatment, the expression of genes associated with the SOS pathway in both the hybrid offspring and their parents was significantly altered, suggesting that the SOS signaling pathway plays a crucial role in response to salt stress. In the comparative analysis between the JX and XT groups, the expression levels of *CmSOS1*, *CmSOS2*, *CmSOS3*, and *CmHKT* genes were significantly decreased in the ‘Yannong Jinxia’ group, whereas the expression level of the 14-3-3 gene was upregulated. This result suggests that the SOS signaling pathway is inhibited, leading to a weakened Na^+^ transport capacity, consequently, a reduced ability of ‘Yannong Jinxia’ to cope with salt stress. In the comparative analysis between the QJ and XT groups, it was found that the gene expression levels of *CmSOS1*, *CmSOS2*, *CmSOS3*, and *CmHKT* in ‘Yannong Qiujin’ were significantly upregulated, whereas the expression level of the 14-3-3 genes was downregulated. This may be achieved by enhancing Na^+^ excretion and Na^+^ unloading in the xylem, thereby improving ‘Yannong Qiujin’’s ability to cope with salt stress.

Through transcriptome sequencing and KEGG analysis, it was shown that metabolic pathways such as plant hormone signal transduction, starch and sucrose metabolism, and phenylpropanoid biosynthesis were significantly enriched in the three comparison groups QJ vs. XT, JX vs. XT, and JX vs. QJ. The Galactose metabolism pathway was specifically enriched in the QJ vs. XT group, while the isoflavone biosynthesis pathway was enriched exclusively in the JX vs. XT group, and the glycerophospholipid metabolism pathway was enriched solely in the JX vs. QJ group. The galactose metabolism pathway plays a critical role in plant salt tolerance through the regulation of osmoprotectant synthesis and cell wall modification. The expression of key genes, such as *GolS* (galactinol synthase) and *RS* (cellobiose synthase), can promote the synthesis of raffinose family oligosaccharides (RFOs) in cotton [49]. These soluble sugars, which function as osmotic regulatory substances, help maintain cellular water balance and stabilize protein structures. In the comparative analysis of QJ vs. XT, the expression level of the *GolS* gene in ‘Yannong Qiujin’ was significantly higher than that in the hybrid offspring, which might contribute to its stronger salt tolerance. Isoflavones exhibit strong antioxidant activity and can effectively scavenge ROS induced by salt stress, thereby alleviating oxidative damage and enhancing the adaptability of plants to high-salt environments. In the hybrid offspring, the expression levels of genes involved in the isoflavone biosynthesis pathway, such as phenylalanine ammonia-lyase (*PAL*) and 4-caffeoyl-CoA ligase (*4CL*), were significantly higher than those in ‘Yannong Jinxia’. The high expression of *PAL* and *4CL* genes promotes the accumulation of isoflavones. Heterologous overexpression of *GmPAL1.1* in *Arabidopsis* reduced H_2_O_2_ accumulation levels and significantly enhanced salt and drought stress resistance in transgenic plants during the germination stage [50]. Glycerophospholipids constitute a major component of cell membranes. Under salt stress, the membrane structure becomes more susceptible to damage. Plants can enhance their salt tolerance by modulating the composition of phospholipids (increasing the proportion of unsaturated fatty acids) and activating *PLD* and *PLC* phospholipases. Researchers discovered that under salt stress, *PeGLABRA3* can bind to the promoter of *AtPLDδ* and activate its transcription [51]. The expression of *AtPLDδ* not only promotes Na^+^ excretion but also reduces the accumulation of ROS, thereby enhancing the salt tolerance of poplar trees. In the JX vs. QJ group, the expression level of the *PLD* gene in ‘Yannong Qiujin’ was significantly higher than that in ‘Yannong Jinxia’. *PLD* is a key phospholipase involved in maintaining membrane fluidity, reducing ionic toxicity, and enhancing salt stress tolerance. Consequently, ‘Yannong Qiujin’ demonstrated superior salt tolerance.

## 5. Conclusions

Collectively, these results demonstrate that viable hybrid progeny were successfully generated in chrysanthemum through intercultivar crossing between an elite ornamental cultivar and a highly salt-tolerant genotype, and that the resulting hybrids exhibited significantly enhanced salt tolerance compared with the male parent. Physiological and transcriptomic analyses were conducted to characterize the salt tolerance phenotype of the hybrid progeny and to identify candidate signaling pathways underlying its regulation. These results enrich the molecular regulatory network of salt stress response in chrysanthemum, laying a theoretical and practical foundation for the innovation of salt-tolerant germplasm resources, breeding of new resistant varieties, and in-depth elucidation of salt tolerance mechanisms.

## Figures and Tables

**Figure 1:**
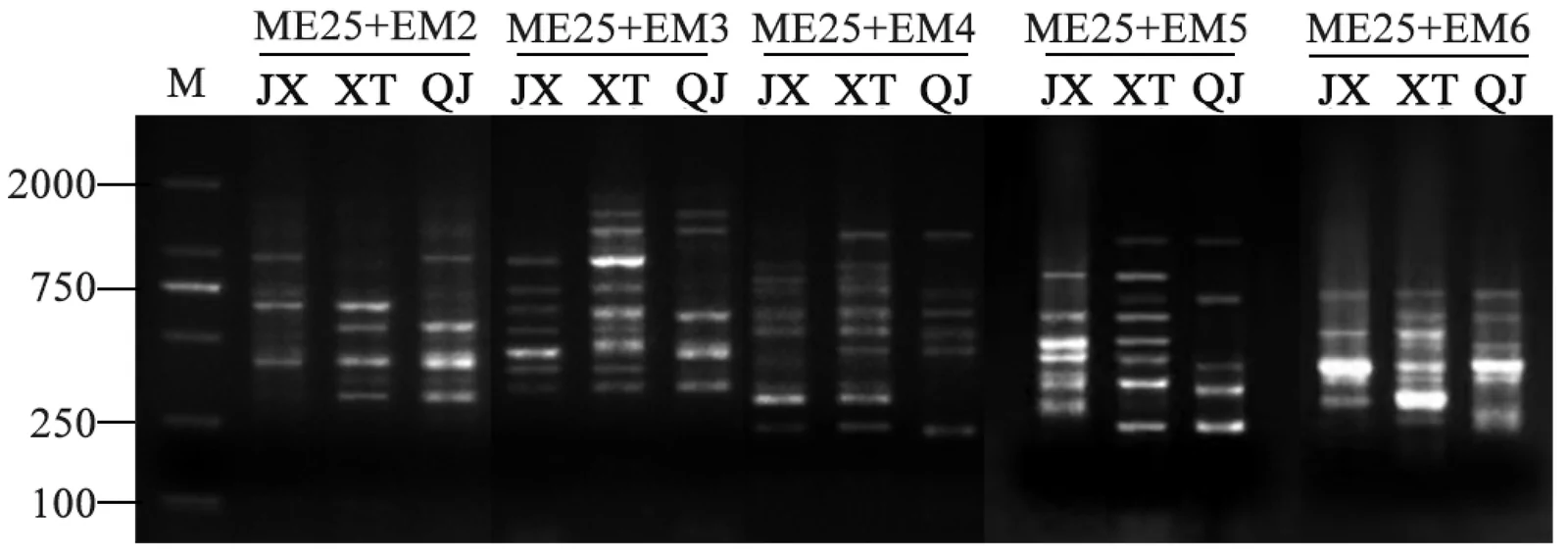
Electrophoresis for identification of hybrid progeny by SRAP-PCR. JX representing ‘Yannong Jinxia’; QJ representing ‘Yannong Qiujin’; XT representing offspring ‘Yannong Qiujin’ × ‘Yannong Jinxia’; ME and EM as a pair of primers; M: marker DNA (2000 bp).

**Figure 2:**
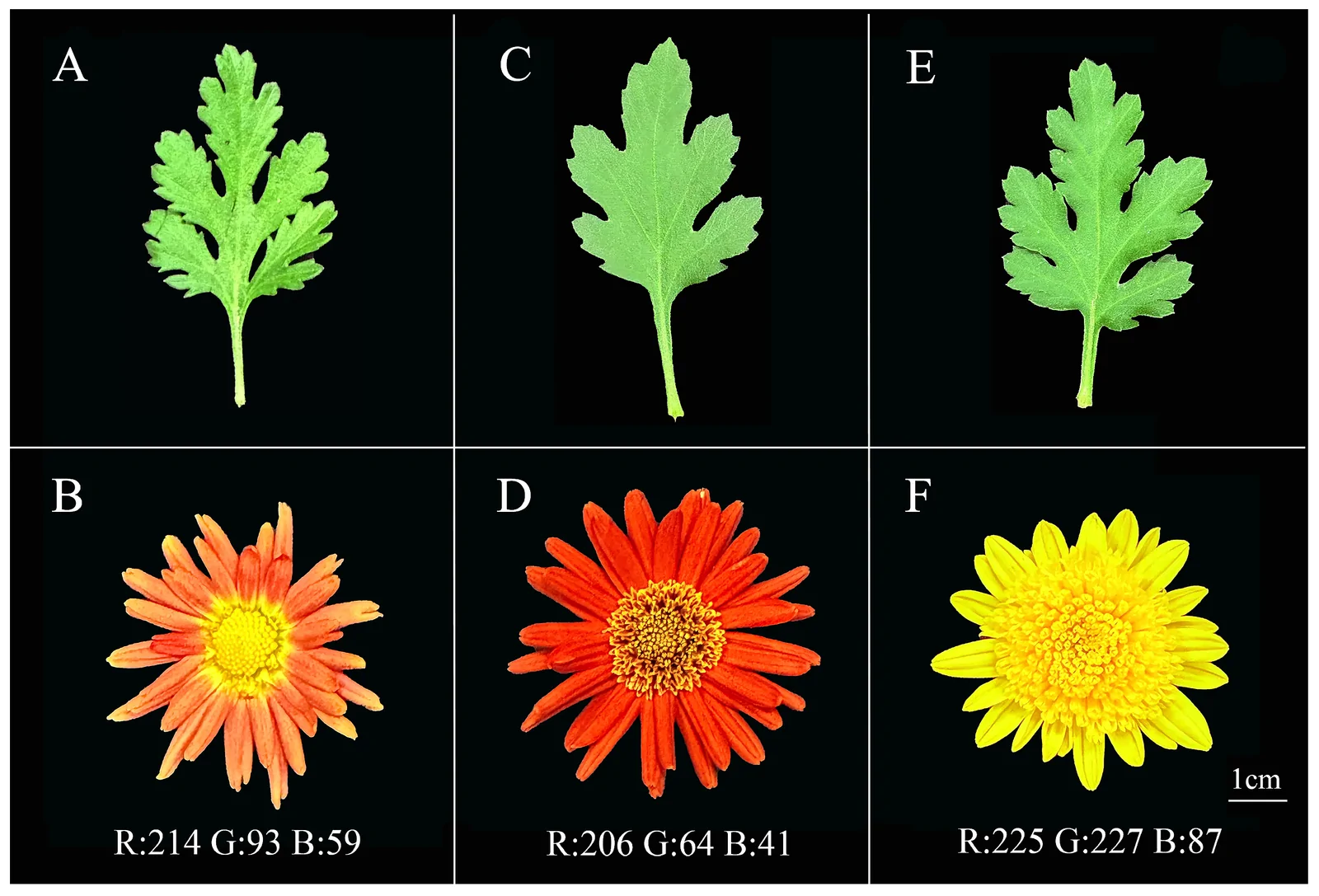
Morphological of leaves and flowers in hybrid progeny and their parental plants. (**A**,**B**) ‘Yannong Jinxia’ (♂), (**C**,**D**) ‘Yannong Xiutang’, (**E**,**F**) ‘Yannong Qiujin’ (♀). The R, G, B values labeled in the figure represent color parameters measured by a digital colorimeter, where R (Red): Intensity of the red channel (higher values indicate more red components); G (Green): Intensity of the green channel; B (Blue): Intensity of the blue channel. Bar = 1 cm.

**Figure 3:**
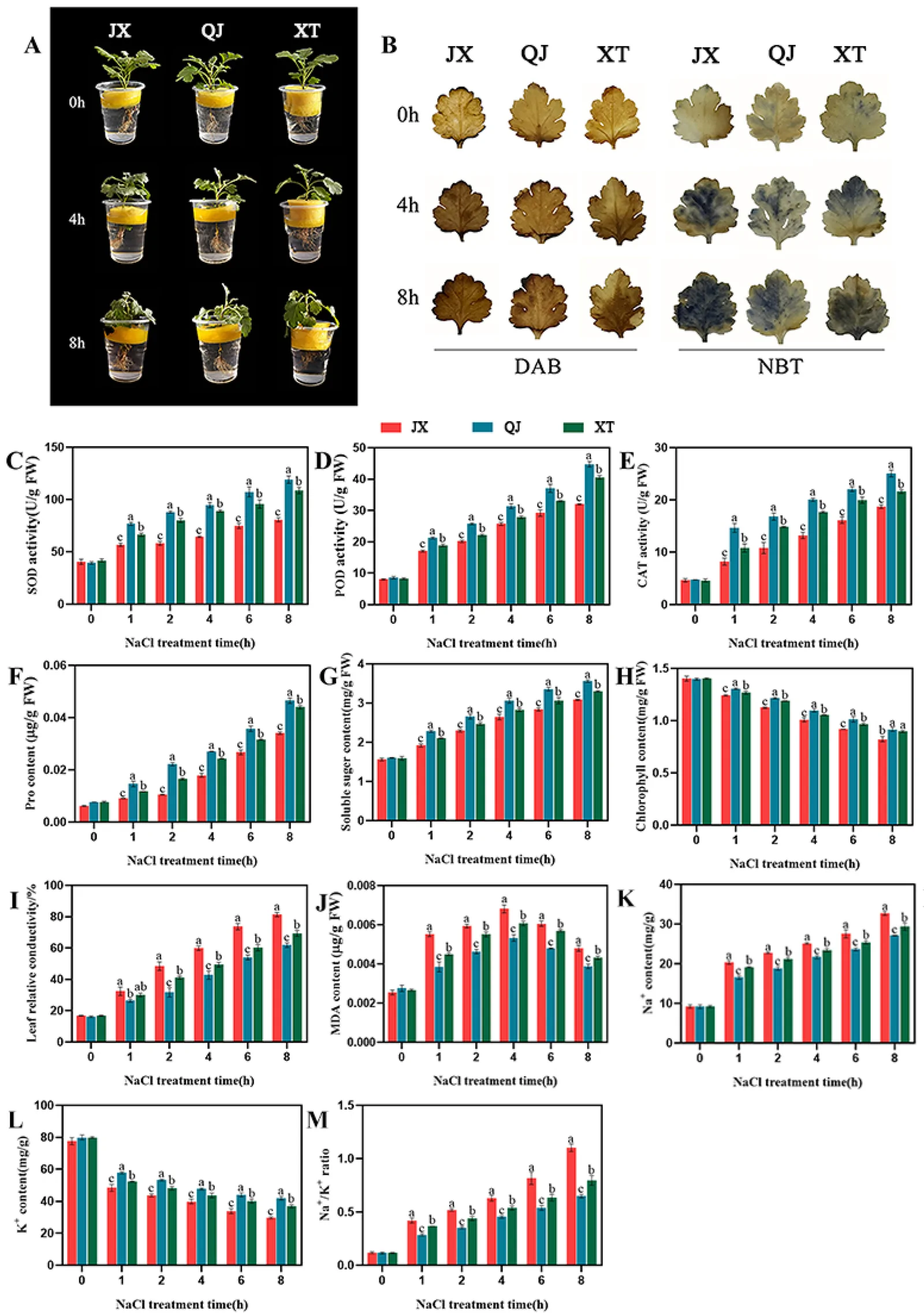
Phenotypic and oxidative responses of hybrid progeny under salt stress. (**A**) Morphological of the hybrid offspring and parental plants under salt stress. (**B**) 3, 3′—Diaminobenzidine (DAB) staining and Nitroblue tetrazolium (NBT) staining. (**C**) Superoxide dismutase (SOD) activity; (**D**) Peroxidase (POD) activity; (**E**) Catalase (CAT) activity; (**F**) Proline (Pro) content; (**G**) Content of soluble suger; (**H**) Content of chlorophyll; (**I**) Relative electrolyte leakage of leaves; (**J**) Malondialdehyde (MDA) content; (**K**) Content of Na+; (**L**) Content of K+; (**M**) Ratio of Na^+^/K^+^. Values are expressed as mean ± SD (n = 3 biological replicates). Different lowercase letters above the bars denote statistically significant differences among genotype, as determined by one-way ANOVA followed by Tukey’s honestly significant difference (HSD) test (*p* < 0.05). JX: Yannong Jinxia (male parent); XT: ‘Yannong Xiutang’ (hybrid progenies); QJ: ‘Yannong Qiujin’ (female parent)’.

**Figure 4:**
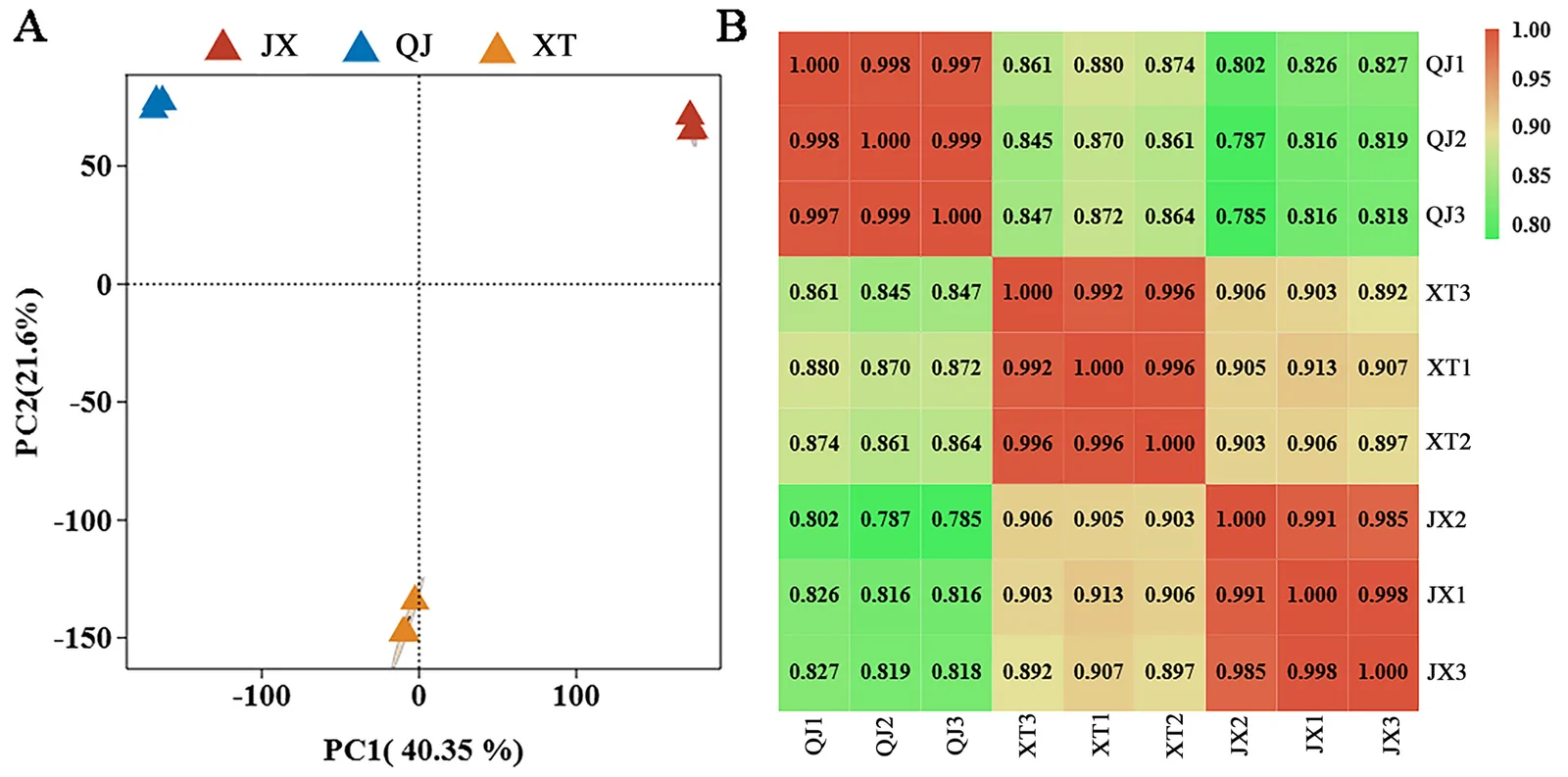
Principal component analysis (**A**) and correlation analysis (**B**) for progeny and parental plants. (**A**) Principal component analysis plot (PCA). PCA was performed on log_2_ (FPKM + 1)-transformed expression values of all expressed genes using the ‘prcomp’ function in R. Prior to analysis, the data were centered and scaled. (**B**) Correlation analysis heat map. Pearson correlation analysis was performed among all sample pairs based on normalized gene expression profiles (FPKM). JX: Yannong Jinxia (male parent); XT: ‘Yannong Xiutang’ (hybrid progenies); QJ: ‘Yannong Qiujin’ (female parent).

**Figure 5:**
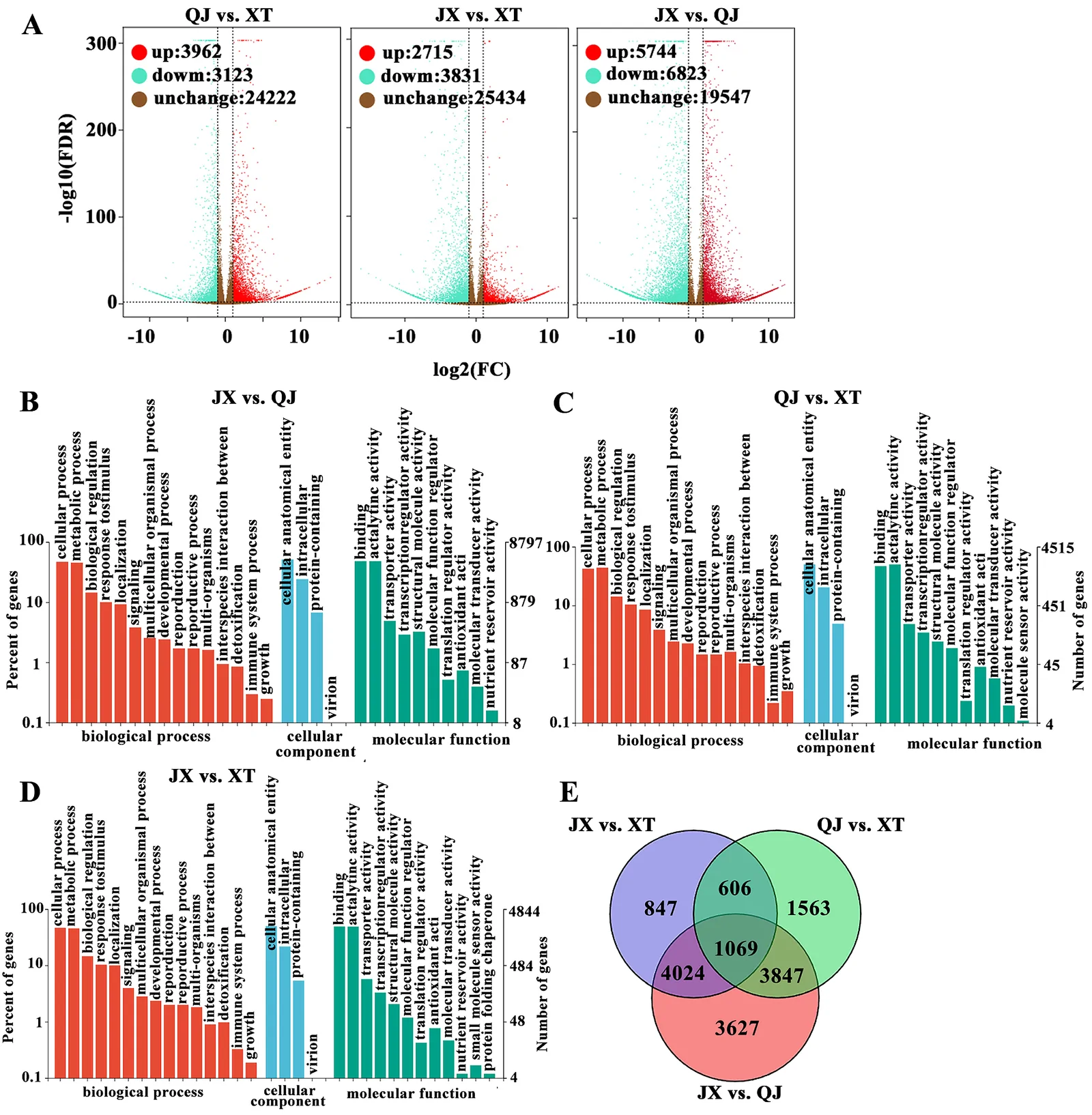
GO enrichment analysis and venn diagram representation of differentially expressed genes. (**A**) Volcano plots of differentially expressed genes. (**B**–**D**) GO enrichment analysis plots. (**E**) Venn diagram. JX: Yannong Jinxia (male parent); XT: ‘Yannong Xiutang’ (hybrid progenies); QJ: ‘Yannong Qiujin’ (female parent). The vertical dashed lines represent |log_2_(fold change)| = 1 (fold change = 2), and the horizontal dashed line represents FDR = 0.01.

**Figure 6:**
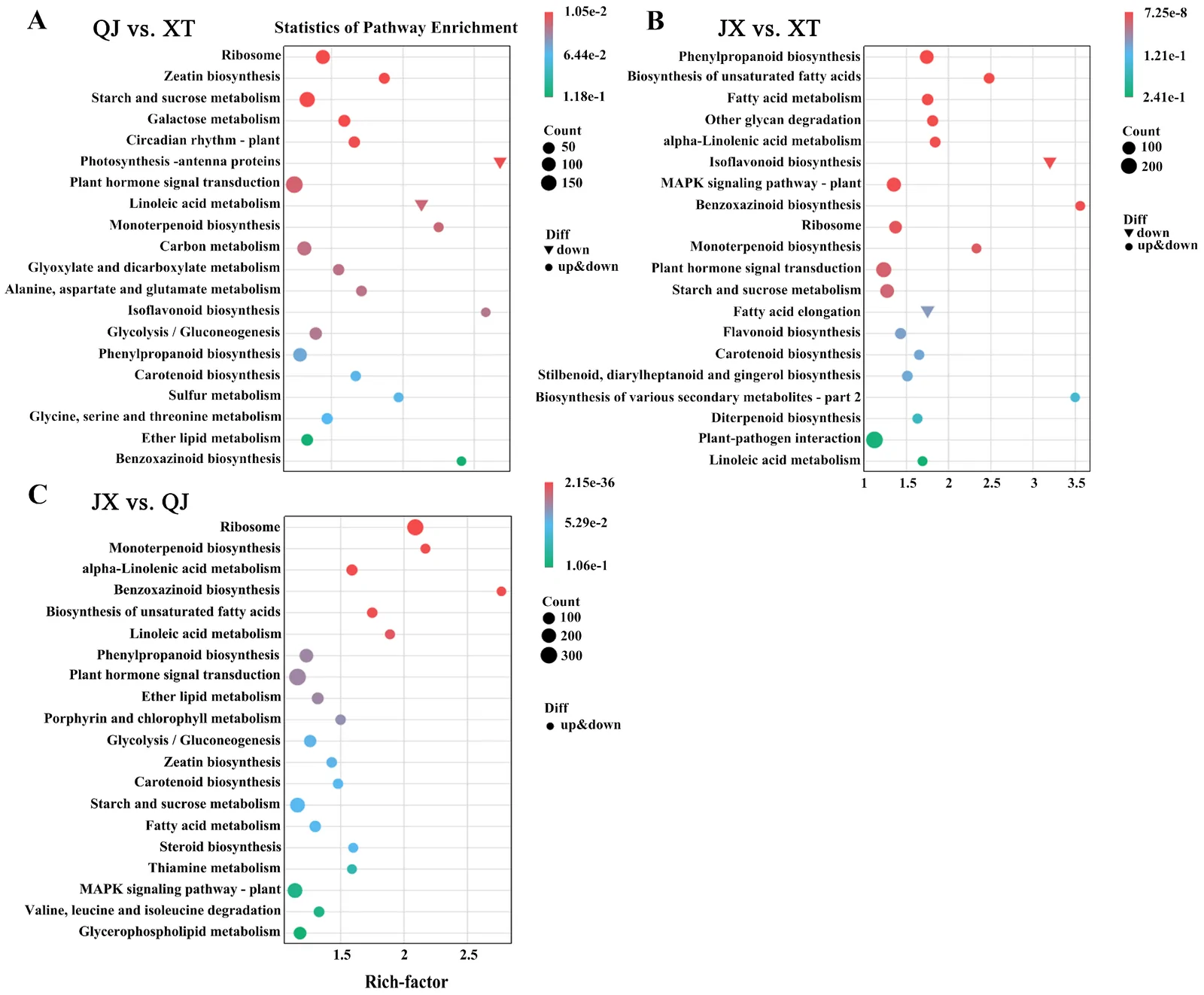
KEGG pathway enrichment analysis of DEGs. (**A**) QJ vs. XT comparisons. (**B**) JX vs. XT comparisons. (**C**) JX vs. QJ comparisons. The analysis revealed the top 20 pathways with the highest enrichment scores. Bubble size and color correspond to the number of genes and the q-value enriched in each pathway, respectively. The apex angle of the triangle indicates. JX: Yannong Jinxia (male parent); XT: ‘Yannong Xiutang’ (hybrid progenies); QJ: ‘Yannong Qiujin’ (female parent).

**Figure 7:**
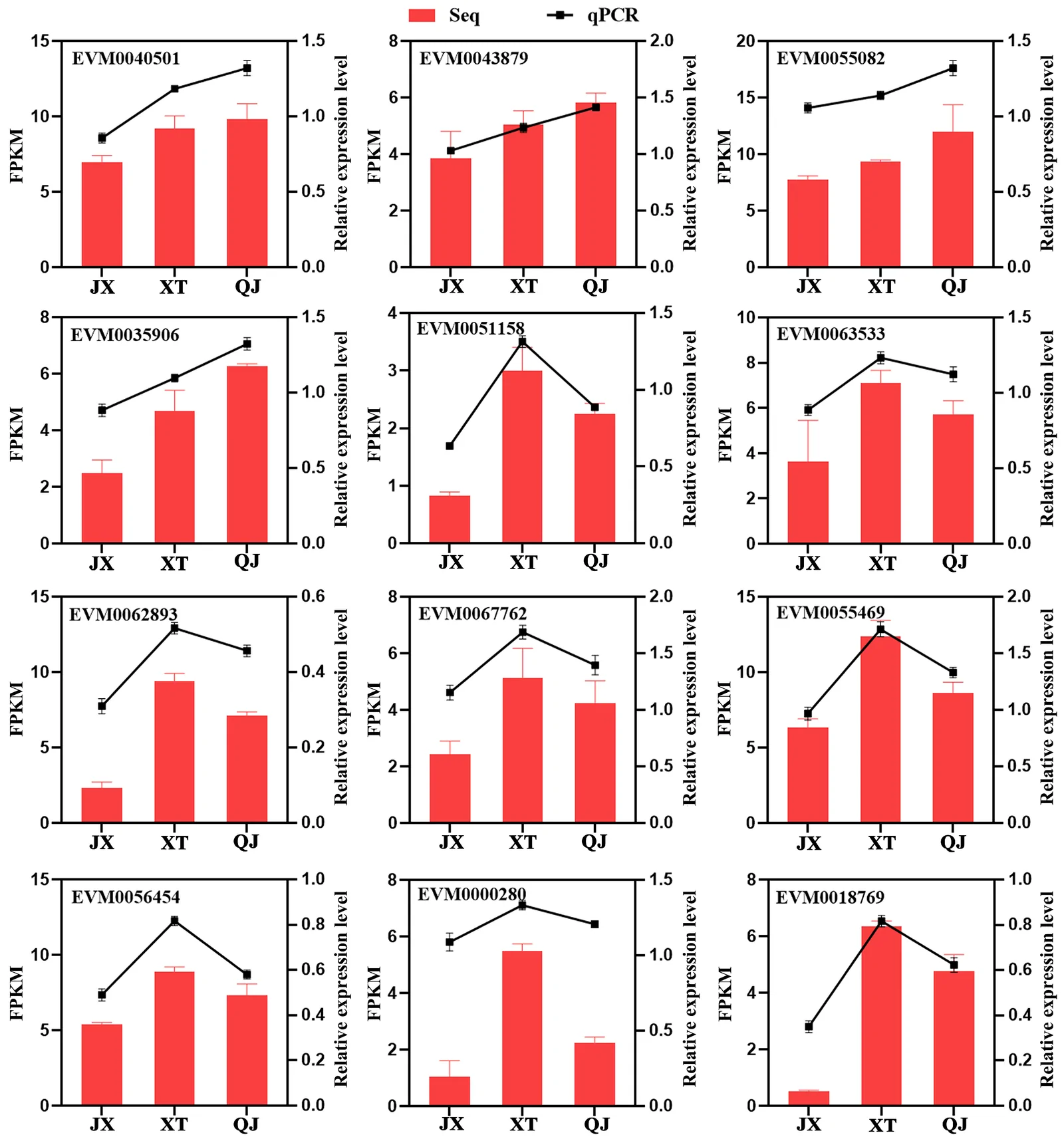
Verification of the expression patterns of RNA-seq results using qRT-PCR. The bar graph spresent the results of the RNA-seq, and the line graphs present the qRT-PCR results. *CmGIP1* (EVM0040501), *CmFRL3* (EVM0043879), *CmXRN4* (EVM0055082), *CmRKF1* (EVM0035906), *CmGAF1* (EVM0051158), *Cm4WRKY70* (EVM0063533), *CmBAK1* (EVM0062893), *CmSAPK3* (EVM0067762), *CmSIS8* (EVM0055469), *CmM3KE1* (EVM0056454), *CmbHLH93* (EVM0000280), *CmNLP7* (EVM0018769). JX: Yannong Jinxia (male parent); XT: ‘Yannong Xiutang’ (hybrid progenies); QJ: ‘Yannong Qiujin’ (female parent).

**Figure 8:**
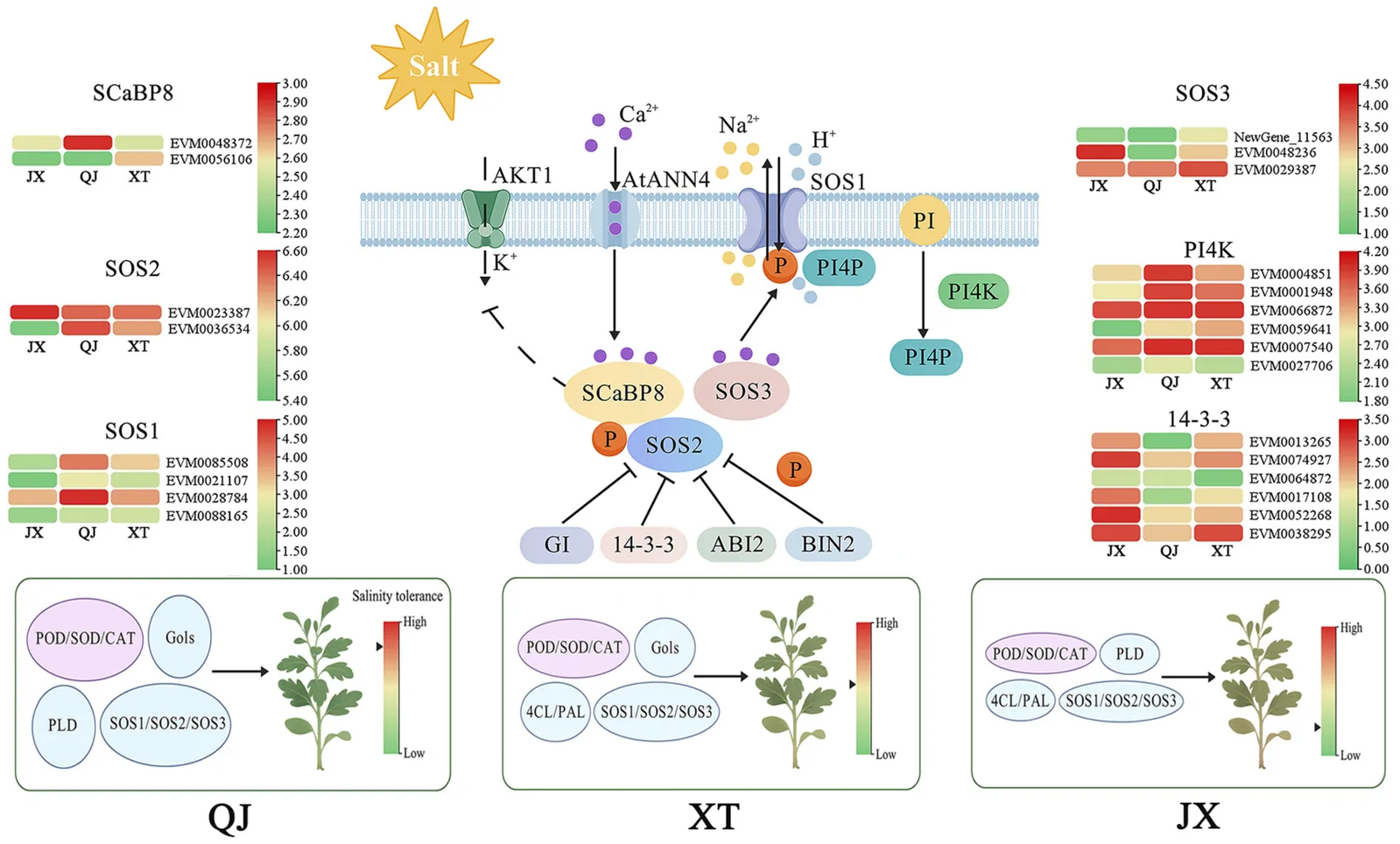
The SOS metabolic pathway and associated gene expression levels. Schematic diagram of salt tolerance acquisition process in QJ, XT, and JX. Red represents high salinity tolerance, green represents low salinity tolerance; the area of the purple circular markers represents the level of key enzyme activity (a larger area indicates higher enzyme activity); the area of the blue circular markers represents the level of key gene expression. Red indicates upregulated expression, green indicates downregulated expression. JX representing ‘Yannong Jinxia’; QJ representing ‘Yannong Qiujin’; XT representing offspring ‘Yannong Jinxia’ × ‘Yannong Qiujin’.

## Data Availability

The data presented in this study are openly available in National Center for Biotechnology Information (NCBI) at https://www.ncbi.nlm.nih.gov/bioproject/, accessed on 23 March 2025, reference number PRJNA1293889. The original contributions presented in this study are included in the article/Supplementary Materials. Further inquiries can be directed to the corresponding authors.

## Supplementary Materials

The following supporting information can be downloaded at: https://www.mdpi.com/article/10.3390/biology15191703/s1, Table S1: Hybrid dominance of traits in the progeny of ‘Yannong Jinxia’ × ‘Yannong Qiujin’; Table S2: SRAP Primer Sequences; Table S3: Primer Sequences; Table S4: Polymorphism analysis; Table S5: Data sequencing was conducted on nine hybrid progeny and their parents; Figure S1: Partial electrophoresis results of hybrid progeny identified by SRAP-PCR; Figure S2: Salt-responsive genes are expressed in under salt stress; Figure S3: Original Western blot images for Figure 1.
