# Supplementary material for: Transcriptome Profiling Reveals the Molecular Regulatory Network in Response to Salt Stress in Intervarietal Hybrids of Chrysanthemum × morifolium ‘Yannong Jinxia’ × ‘Yannong Qiujin’
Source: Biology (Basel). 2026 Sep 24;15(19):1703. doi: 10.3390/biology15191703 (PMC13663464; doi:10.3390/biology15191703)
Supplement: Supplementary file 1 [file biology-15-01703-s001.zip › biology-4534876-supplementary.pdf]

Table S1 Hybrid dominance of traits in the progeny of 'Yannong Jinxia' × 'Yannong Qiujin'

Trait	Yannong Jinxia	Yannong Qiujin	Mid-parent value (MPV)	Hybrid progeny		
				F1 mean F_m	Mid-parent Heterosis H_m	Mid-parent Heterosis rate RH_m (%)
Leaf length (cm)	6.34[a]	6.29[b]	6.31	5.85[c]	-0.46	-7.25
Leaf width (cm)	3.40[b]	4.29[a]	3.84	3.15[c]	-0.69	-18.00
Plant height (cm)	21.67[c]	33.50[b]	27.58	36.83[a]	9.25	33.53
Crown diameter (cm)	43.5[c]	50.00[b]	46.75	55.50[a]	8.75	18.72
Diameter of inflorescence (cm)	4.51[b]	4.20[c]	4.36	5.47[a]	1.11	25.50
Number of ray flowers	124.00[b]	177.67[a]	150.83	120.50[c]	-30.33	-20.11
Length of ray flowers (cm)	0.53[c]	1.24[a]	0.89	0.83[b]	-0.06	-6.59
Number of tubular flower	26.33[b]	22.33[c]	54.33	33.17[a]	8.83	36.30
Length of tubular flower (cm)	2.08[b]	1.86[c]	1.97	2.38[a]	0.41	21.05

Lowercase letters indicating significant differences

Table S2 SRAP Primer Sequences

Primer	Sequence (5′–3′)	Primer	Sequence (5′–3′)
EM2	GACTGCGTACGAATTTGC	ME4	TGAGTCCAAACCGGACC
EM3	GACTGCGTACGAATTGAC	ME6	TGAGTCCAAACCGGTAG
EM4	GACTGCGTACGAATTTGA	ME8	TGAGTCCAAACCGGTGT
EM5	GACTGCGTACGAATTAAC	ME10	TGAGTCCAAACCGGATG
EM6	GACTGCGTACGAATTGCA	ME23	CACAGTCATGCCGGAAT
EM11	GACTGCGTACGAATTTCG	ME25	CAGGACTAAACCGGATA
EM12	GACTGCGTACGAATTGTC		
EM14	GACTGCGTACGAATTCAG		
EM18	GACTGCGTACGAATTGAT		

Table S3 Primer Sequences

Primer name	Sequence (5′–3′)
qRT-*CmEF1α*-F	CCATTCAAGCGACAGACTCA
qRT-*CmEF1α*-R	TTTTGGTATCTGGTCCTGGAG
qRT-*CmGIP1*-F	ATAATAGGACGCAACAAGGGAT
qRT-*CmGIP1*-R	GGATGATAATGGGACAGGTGA
qRT-*CmFRL3*-F	TAGGATTGCCTCAGAGGTTGG
qRT-*CmFRL3*-R	GATTGTGGGCTGGTTTGGTAG
qRT-*CmXRN4*-F	TGAGTTGTTGAAACCTAACCCTA
qRT-*CmXRN4*-R	ATAACCACCCATTGCAGTAAA
qRT-*CmRKF1*-F	CATTGGATAAAGCCACAGGAA
qRT-*CmRKF1*-R	GGAACACTCGTATTTGGGACA
qRT-*CmGAF1*-F	AGAGTTATGATTTAGTTGCGGTTAG
qRT-*CmGAF1*-R	ATTCAAAGAGCCACGAGACAA
qRT-*CmWRKY70*-F	GAGATGGTTATTCGTGGAGGA
qRT-*CmWRKY70*-R	GGCGTCACTTCGGTTATTTGT
qRT-*CmBAK1*-F	GAACCTGAACCCTCCACCTCC
qRT-*CmBAK1*-R	AACTGTAACTCGCCACCTTGA
qRT-*CmSAPK3*-F	ACAAAGGAAACGGAAGGAGGT
qRT-*CmSAPK3*-R	GTTCATCAATGGTGGTGGAGC
qRT-*CmSIS8*-F	TAATGAGGCTACGAGTGGTGA
qRT-*CmSIS8*-R	CAATAATACTTGCGACTTGTGA
qRT-*CmM3KE1*-F	TTGGAGATGAGATTGGGAAAG
qRT-*CmM3KE1*-R	AGGTGATACCATGTGCGACAG
qRT-*CmbHLH93*-F	TGACTTACTACTGTTGCCACCTT
qRT-*CmbHLH93*-F	CAAACATCTGCCTCCATAACC
qRT-*CmNLP7*-F	ATGCCTATTTATTGTCCTTCC
qRT-*CmNLP7*-F	TCACAGCCACTTGTTTATTCC

Table S4 Polymorphism analysis

Prime name	Shannon information index	Nei gene diversity index	percentage of polymorphic loci
ME25+EM2	0.6524	0.4602	100%
ME25+EM3	0.4427	0.3143	66.67%
ME25+EM4	0.4484	0.3198	66.67%
ME25+EM5	0.4326	0.3047	66.67%
ME25+EM6	0.6326	0.4415	100%
ME8+EM11	0.4451	0.3166	66.67%
ME25+EM18	0.4241	0.2963	66.67%
ME8+EM17	0.6407	0.3387	100%
ME25+EM6	0.4575	0.3288	66.67%
ME25+EM11	0.6661	0.4733	100%
ME8+EM18	0.4584	0.3296	66.67%
ME8+EM3	0.6247	0.4353	100%
ME8+EM4	0.4487	0.3201	66.67%

Table S5 Data sequencing was conducted on nine hybrid progeny and their parents.

Samples	Clean reads	GC Content	%≥Q30	Total Reads	Mapped Reads
JX1	21,426,458	43.44%	95.43%	42,852,916	25,275,600 (58.98%)
JX2	21,872,333	43.58%	95.41%	43,744,666	26,168,809 (59.82%)
JX3	21,113,365	43.01%	95.23%	42,226,730	24,685,826 (58.46%)
QJ1	22,179,667	43.98%	97.20%	44,359,334	27,433,239 (61.84%)
QJ2	22,168,291	43.74%	97.08%	44,336,582	27,840,762 (62.79%)
QJ3	20,661,458	43.63%	97.10%	41,322,916	25,806,842 (62.45%)
XT1	22,954,819	43.52%	95.75%	45,909,638	28,191,059 (61.41%)
XT2	22,458,572	43.54%	95.64%	44,917,144	27,605,133 (61.46%)
XT3	21,992,068	43.55%	95.88%	43,984,136	27,087,447 (61.58%)

JX representing ‘Yannong Jinxia’; QJ representing ‘Yannong Qiujin’; XT representing offspring ‘Yannong Jinxia’× ‘Yannong Qiujin’

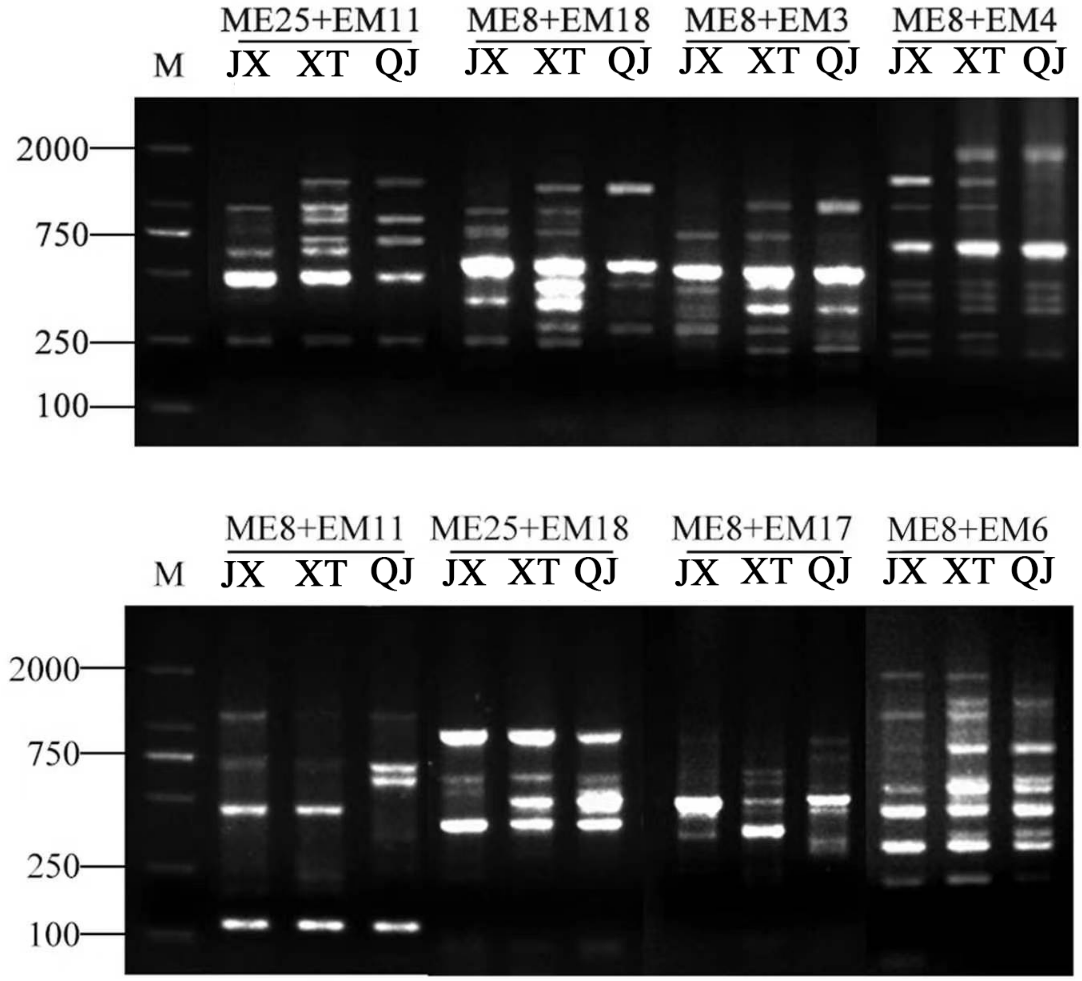

Figure S1 Partial electrophoresis results of hybrid progeny identified by SRAP-PCR. JX representing ‘Yannong Jinxia'; QJ representing ‘Yannong Qiujin'; XT representing offspring ‘Yannong Jinxia' × ‘Yannong Qiujin'; ME and EM as a pair of primers; M: marker DNA (2000 bp).

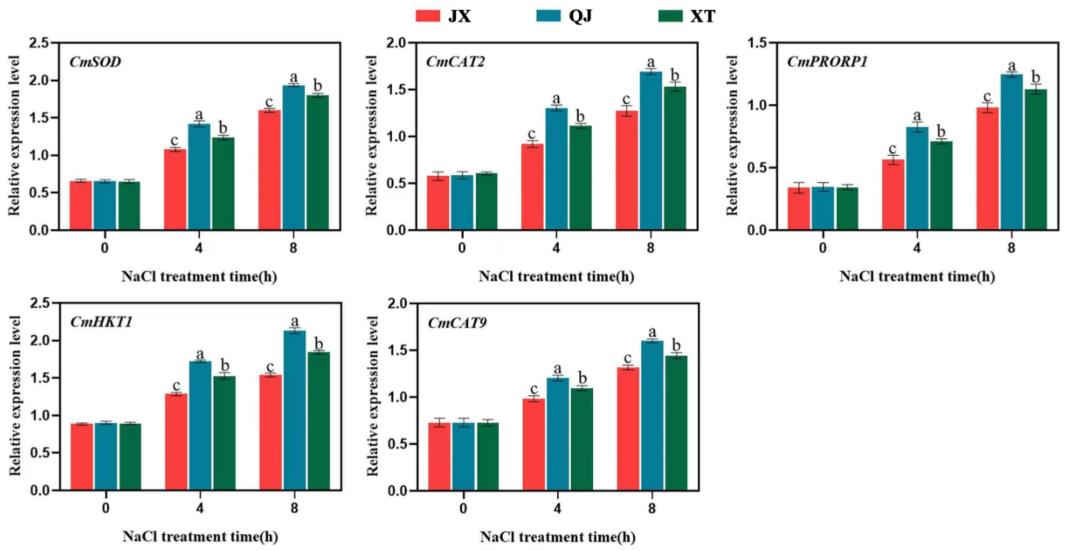

Figure S2 Salt-responsive genes are expressed in under salt stress.

JX representing 'Yannong Jinxia'; QJ representing 'Yannong Qiujin'; XT representing offspring 'Yannong Jinxia' × 'Yannong Qiujin'.

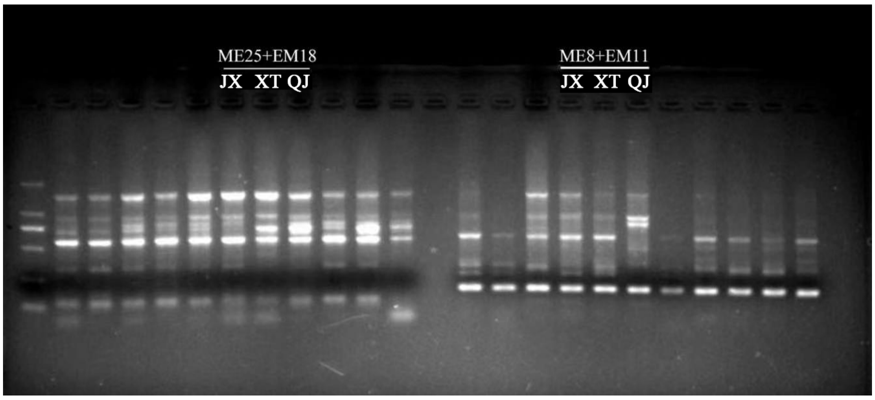
ME25+EM18
JX XT QJ
ME8+EM11
JX XT QJ

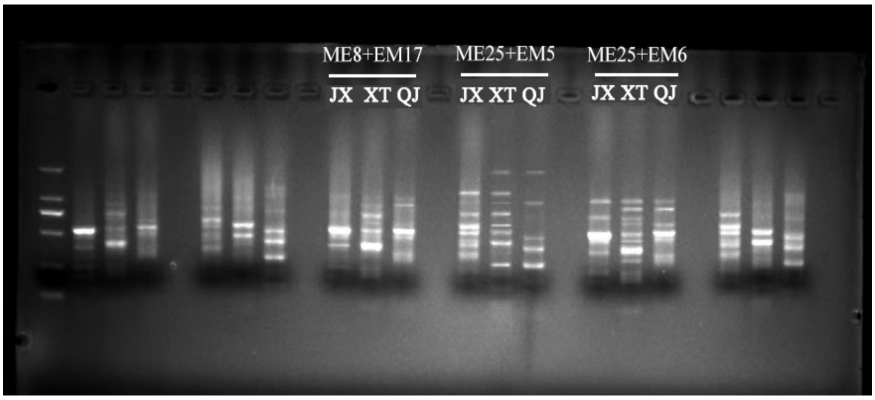
ME8+EM17
JX XT QJ
ME25+EM5
JX XT QJ
ME25+EM6
JX XT QJ

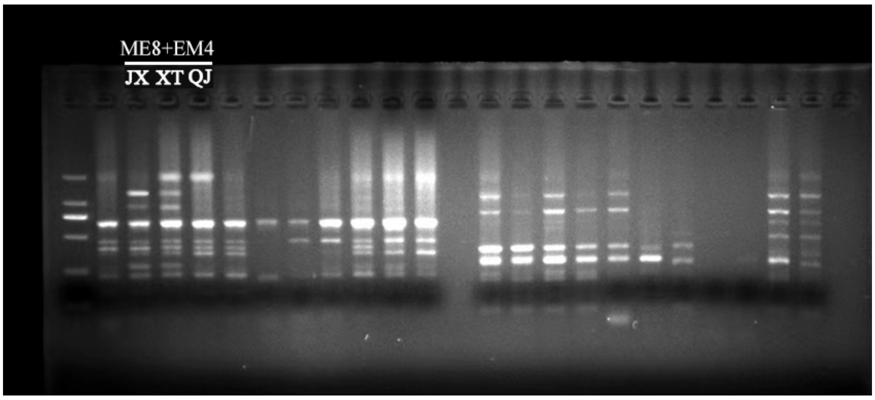
ME8+EM4
JX XT QJ

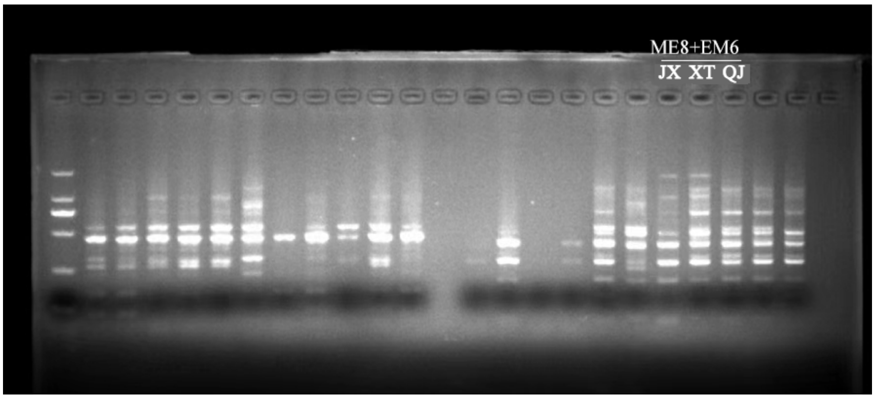
ME8+EM6
JX XT QJ

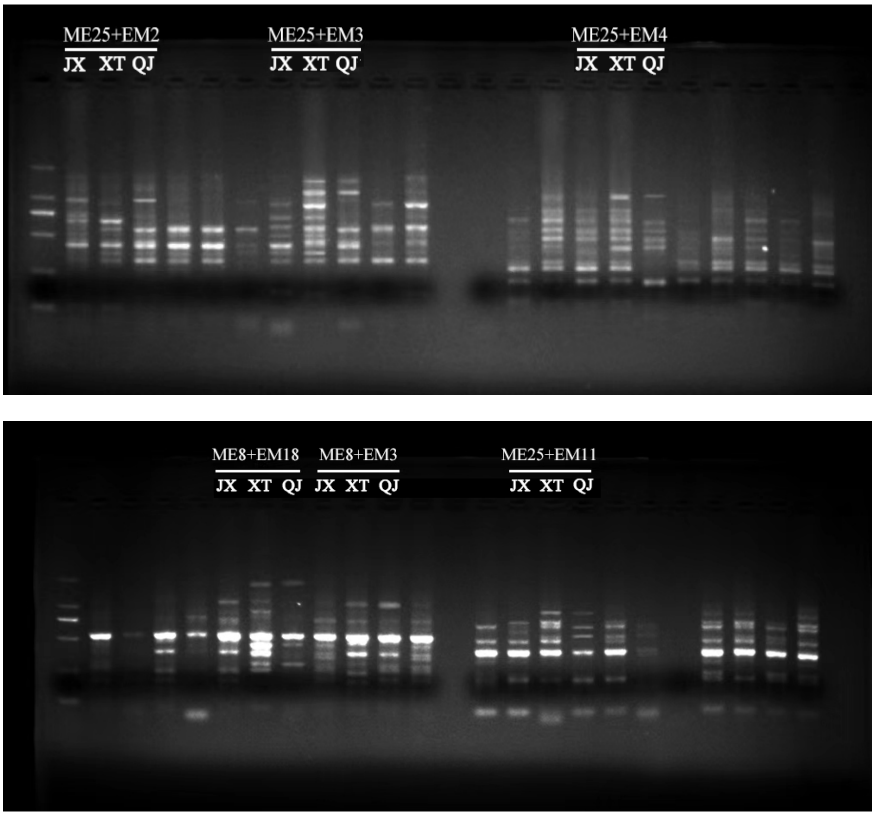

Figure S3 Original Western blot images for Figure 1.
